# Dietary and Lifestyle Management of Functional Hypothalamic Amenorrhea: A Comprehensive Review

**DOI:** 10.3390/nu16172967

**Published:** 2024-09-03

**Authors:** Katarzyna Dobranowska, Stanisława Plińska, Agnieszka Dobosz

**Affiliations:** 1 Division of Basic Medical Sciences, Department of Basic Medical Sciences and Immunology, Wroclaw Medical University, Borowska 211 Str., 50-556 Wrocław, Poland; 2 Department of Basic Chemical Sciences, Wroclaw Medical University, Borowska 211a Str., 50-556 Wrocław, Poland

**Keywords:** functional hypothalamic amenorrhea (FHA), dietary intervention, energy availability, energy deficit, nonpharmacological treatment of FHA

## Abstract

Functional Hypothalamic Amenorrhea (FHA) is a condition characterized by the absence of menstruation, which is increasingly affecting young women. However, specific recommendations for treating and preventing this condition are lacking. Based on a review of the available literature, this article provides practical and feasible dietary management recommendations for healthcare professionals and researchers in women’s health and nutrition. It answers the question of what interventions and nutritional recommendations are necessary to restore menstrual function in women struggling with FHA. Physicians recommend an energy availability threshold of 30 kcal/kg FFM/day to prevent FHA. Also, energy availability below and above this threshold can inhibit LH pulsation and cause menstrual disorders. In addition, the risk of menstrual disorders increases with a decrease in the caloric content of the diet and the duration of the energy deficit, and women with FHA have significantly lower energy availability than healthy women. It is essential to ensure that adequate kilocalories are provided throughout the day (regular meals that are a source of proper glucose) to avoid a negative energy balance, as glucose has been proven to affect LH pulses and T3 and cortisol concentrations in the body. Dietary intervention should focus on increasing the caloric content of the diet, thus increasing energy availability and restoring energy balance in the body. Treatment and diagnosis should also focus on body composition, not just body weight. An increase in body fat percentage above 22% may be required to restore menstrual function. In women with FHA, even an increase in body fat mass of one kilogram (kg) increases the likelihood of menstruation by 8%. It is advisable to reduce the intensity of physical activity or training volume, while it is not advisable to give up physical activity altogether. It is also important to ensure adequate intake of micronutrients, reduce stress, and incorporate cognitive–behavioral therapy.

## 1. Introduction

Functional hypothalamic amenorrhea (FHA) is a common problem among physically active women, especially in sports related to body shape and endurance. However, the problem is also increasingly prevalent in women playing sports recreationally and aiming for weight loss [1,2]. Long-term persistent FHA increases the risk of osteoporosis, cardiovascular disease, depression, and infertility [3,4]. It is, therefore, essential to raise awareness of the FHA problem and identify appropriate treatments.

Functional hypothalamic amenorrhea is defined as the absence of menstruation for a period of three or six months in a previously menstruating woman. It is associated with the inhibition of the hypothalamic–pituitary–ovarian (HPO) axis [2,3,4,5]. Three main factors are assumed to cause functional hypothalamic amenorrhea: psychological stress, caloric restriction, and excessive physical activity. Unlike other causes of secondary amenorrhea, such as prolactinoma, which is also reversible but typically through medical or surgical treatment, FHA is reversible by correcting or improving the contributing lifestyle factors [6]. The cerebral cortex, the hypothalamus, the anterior gland of the pituitary gland, and the ovaries influence the regulation of the reproductive system [3]. Hormonal changes are closely linked to the menstrual cycle, and any stress, as well as environmental, psychological, and social factors perceived by the brain, can affect and disrupt it [2]. Certain brain regions and pathways play critical roles in controlling the hypothalamus. For instance, the pro-opiomelanocortin (POMC) and cocaine–amphetamine-regulated transcript (CART) neurons promote satiety, while neuropeptide Y (NPY) and agouti-related peptide (AgRP) neurons stimulate appetite. Additionally, corticotropin-releasing hormone (CRH) pathways are involved in the stress response, which can impact the HPG axis and menstrual function. Dopamine also plays a significant role by inhibiting prolactin secretion and modulating GnRH secretion through its action on the hypothalamus and pituitary gland [3]. The hypothalamus produces GnRH (gonadotropin-releasing hormone), which controls the secretion of LH (luteinizing hormone) and FSH (follicle-stimulating hormone) [3]. LH and FSH, which are pulsatile secreted from the anterior pituitary gland, control the production of ovarian steroid hormones (estradiol, progesterone, and androgens) and ovulation. Negative feedback from ovarian hormones acts on the anterior pituitary gland to stop the secretion of LH and FSH [3]. Estrogen acts in mid-cycle positive feedback, increasing the secretion of GnRH, LH, and FSH. In addition to steroid hormones, the ovaries also produce the peptide hormones inhibin and follistatin, which inhibit FSH secretion, and activin, which increases FSH secretion [3]. It is worth noting that the feedback mechanisms are very complex and vary according to many factors. They are finely regulated in the body to maintain hormonal homeostasis and ensure proper endocrine function [3].

**Figure 1:**
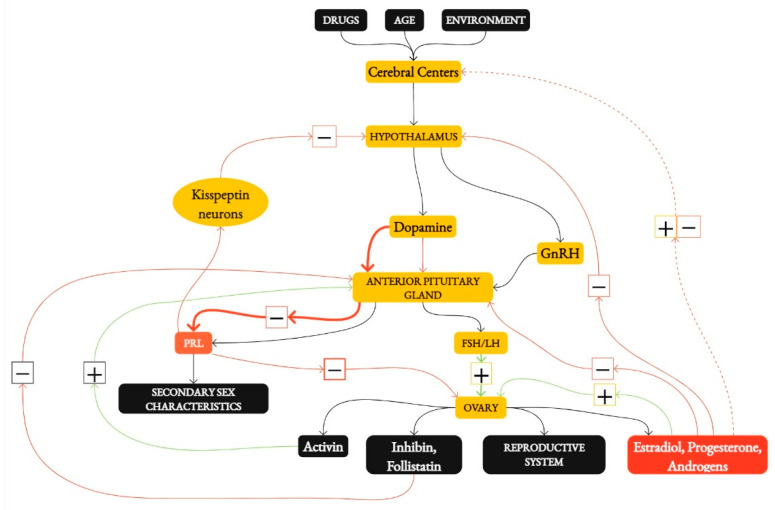
Regulation of the Reproductive System. Abbreviations: GnRH—Gonadotropin-Releasing Hormone, LH—Luteinizing Hormone, FSH—Follicle-Stimulating Hormone, PRL—Prolactin.

Figure 1 illustrates the intricate regulatory pathways of the reproductive system. It highlights the roles of the cerebral cortex, hypothalamus, pituitary gland, and ovaries in controlling the secretion of essential hormones such as GnRH, LH, and FSH. The feedback loops involving estrogen and other ovarian hormones are also depicted, showcasing how these elements work together to maintain hormonal balance and reproductive function [3].

Studies have shown that GnRH secretion is inhibited in patients with hypothalamic amenorrhea, reducing LH and FSH secretion to a level where they cannot support folliculogenesis (the developmental process of ovarian follicles) [1,2,3]. A consequence of the disruption of the pulsatile secretion of GnRH and gonadotropins is a low concentration of estrogen, so-called hypoestrogenism. This state harms women’s health, as estrogen is essential for normal bone metabolism and cardiovascular and mental health [5,7]. The mechanism responsible for FHA is not entirely understood. What is certain is that numerous neurotransmitters and neurosteroids are involved in regulating the hypothalamic–pituitary–ovarian axis and, thus, in the pathophysiology of FHA. The most important appear to be kisspeptin, leptin, beta-endorphin, neuropeptide Y (NPY), ghrelin, and corticotrophin-releasing hormone (CRH) [8]. Kisspeptin is crucial for GnRH secretion and reproductive function. Leptin, secreted by adipose tissue, signals energy sufficiency and influences reproductive hormones. Beta-endorphin, an endogenous opioid, can suppress GnRH secretion. Neuropeptide Y (NPY) stimulates appetite and can negatively affect reproductive function. Ghrelin, a hunger hormone, inhibits GnRH release, and CRH, which is involved in the stress response, can also inhibit reproductive function through its action on the hypothalamus [3,8].

Higher cortisol levels are also frequently observed in women with FHA. This phenomenon and associated mechanisms suggest a specific role for stress in the etiology of hypothalamic amenorrhea [7].

Patients with FHA also have increased levels of ghrelin, an appetite-stimulating hormone. This may be related to metabolic adaptation resulting from chronic energy deprivation and activation of the appetite-stimulating mechanism. Higher ghrelin concentrations may interfere with GnRH secretion, lead to hypogonadism, and prolong FHA in patients who have regained a normal body weight [7].

FHA is associated with significantly reduced leptin levels, a hormone secreted primarily by adipocytes in white adipose tissue and enterocytes. Its secretion increases as stored adipose tissue increases, suppressing appetite and enhancing energy expenditure. Leptin achieves appetite suppression by promoting the release of pro-opiomelanocortin (POMC) and inhibiting the release of neuropeptide Y (NPY) and agouti-related peptide (AGRP) from the arcuate nucleus of the hypothalamus [3,9]. In conditions like FHA, where energy intake is insufficient, leptin levels drop significantly. Leptin concentrations are directly related to nutritional status, and when leptin levels fall below a critical threshold, menstruation does not occur. This is because leptin is involved in signaling the hypothalamus to initiate the reproductive hormone cascade necessary for ovulation and menstruation [10,11]. FHA also impacts thyroid function and is associated with a decline in thyroid hormone levels. The thyroid gland produces hormones like thyroxine (T4) and triiodothyronine (T3), which regulate metabolism, including basal metabolic rate (BMR). In FHA, reduced energy intake and stress lead to a decrease in leptin, which in turn reduces the stimulation of the thyroid axis. This results in lower levels of thyroid hormones, further contributing to a reduced BMR often observed in non-menstruating, exercising women [7,12].

## 2. Methods and Aim of Work

A comprehensive literature review was conducted to investigate the impact of diet and physical activity on the menstrual cycle, mainly focusing on the dysfunction of the hypothalamic–pituitary–ovarian axis. The review targeted scientific papers and books published between 2013 and 2023 to ensure the inclusion of the most current and relevant data. This timeframe was chosen to capture recent advancements and findings in the field, though the review occasionally referenced seminal older studies to provide necessary context.

The literature search was performed using several electronic databases, including PubMed, EBSCO, Scopus, and Google Scholar. The primary keywords employed in these searches were “Functional hypothalamic amenorrhea (FHA),” “diet and menstrual cycle,” “physical activity and menstrual cycle,” “low energy availability,” “hypothalamic-pituitary-ovarian axis dysfunction,” “sports-related menstrual disorders,” and “energy balance and endocrine function.” These keywords were used in various combinations to ensure a comprehensive coverage of the relevant literature.

Included in the review were studies published between 2013 and 2023, with a particular emphasis on the most recent research within this timeframe. Only peer-reviewed scientific papers and books were considered, focusing on research that examined the relationship between diet, physical activity, and menstrual function, as well as studies that explored interventions and dietary recommendations for restoring menstrual function in women experiencing hypothalamic amenorrhea.

Data extracted from the selected studies included the prevalence of hypothalamic amenorrhea among physically active women, energy availability and nutritional intake in maintaining normal endocrine function, and adequate dietary and physical activity interventions for regaining menstrual function. This approach allowed for a detailed analysis of how diet and physical activity influence the menstrual cycle, with a particular focus on hypothalamic amenorrhea.

While the review’s primary focus was on studies from the last decade, the inclusion of crucial older studies where necessary provided a foundational understanding. However, the overall emphasis remained on incorporating the latest findings to reflect current scientific consensus and advancements. This methodical approach ensured that the review was based on the most up-to-date and relevant studies, providing a solid foundation for the analysis and findings presented in this paper.

This review aims to bridge the gap between textbook knowledge and healthcare providers’ practical challenges in managing FHA. By synthesizing recent research findings and translating them into practical dietary and lifestyle guidelines, this review seeks to standardize care while emphasizing the need for individualized treatment plans. This balance between standardization and individualization is a critical contribution to the field.

## 3. Results

### 3.1. The Impact of Energy Availability on the Menstrual Cycle

Energy availability is a critical concept in understanding how the body allocates resources, particularly regarding reproductive function. Energy availability refers to the amount of dietary energy remaining for the body’s physiological functions after subtracting the energy expended during exercise [1]. Energy availability strongly correlates with the frequency of LH pulses, their amplitude, and the incidence of menstrual disorders [1]. A lower frequency of LH (luteinizing hormone) pulses means less frequent stimulation of the ovaries. This reduction in stimulation can lead to irregular or absent ovulation (anovulation), as the consistent release of LH is crucial for the maturation and release of the egg during the menstrual cycle. In the context of oligo/anovulation, fewer LH pulses mean fewer opportunities for the ovary to receive the signals necessary for egg release. Higher amplitude pulses indicate a more pronounced release of LH, but this occurs less frequently. The infrequent but intense LH pulses may disrupt the hormonal balance required for the regular menstrual cycle. Increased amplitude without appropriate frequency can contribute to conditions like oligo/amenorrhea, where menstrual cycles are irregular or absent [1,6,13].

A study by Loucks et al. [1] published in 2003 showed a significant effect of energy availability on the pulsatility of luteinizing hormone (LH), an essential hormone regulating the menstrual cycle. At an energy availability of 30 kcal/kg FFM (free fat mass)/day, no significant changes in the frequency of LH pulses were observed in regularly menstruating women. However, when energy availability dropped below this value, the frequency of LH pulses decreased. Reducing energy availability to 20 kcal/kg FFM/day resulted in a 16% reduction in LH pulse frequency and a 21% increase in LH pulse amplitude. In contrast, a decrease in energy availability to 10 kcal/kg FFM/day led to a 39% decrease in LH pulse frequency and a 109% increase in pulse amplitude. The study showed that LH pulse frequency was significantly impaired at an energy availability threshold of less than 30 kcal/kg FFM/day [1].

In contrast to the study by Loucks et al. [1], the survey by Koltun et al. [14] did not show an unambiguous threshold of energy availability from which LH pulse frequency decreases rapidly. However, it is presumed that more significant decreases in energy availability could lead to more severe and frequent menstrual disorders [14].

Similarly, Lieberman et al. [2] found no discernible threshold of energy availability below which menstrual disorders were initiated. When energy availability fell below 30 kcal/kg FFM/day, the probability that the women studied experienced menstrual disorders exceeded 50%. Menstrual disorders also occurred when energy availability was above 30 kcal/kg FFM/day. The most common menstrual disorders were luteal phase defects (57%), lack of ovulation (28%), and oligomenorrhea cycles (15%) [2]. Luteal phase defects occur when the corpus luteum fails to produce adequate progesterone after ovulation, resulting in an endometrium that is not fully prepared for embryo implantation and growth. This can lead to issues such as infertility, early pregnancy loss, and menstrual irregularities, although the diagnosis and clinical relevance of luteal phase defects remain contentious due to the variability of progesterone levels and the lack of reliable diagnostic methods [15]. This study is critical because it challenges the findings of Loucks et al. [1], who suggest that there is a threshold of energy availability (30 kcal/kg FFM/day) below which menstrual disorders occur [1].

Additionally, a study by Reed et al. [10] showed that a threshold of 30 kcal/kg FFM/day was not sufficient to distinguish between regularly menstruating women and women characterized by menstrual disorders. Instead, energy availability (EA) was significantly lower in the group of women with FHA than in the periodically menstruating women (30.9 ± 2.4 vs. 36.9 ± 1.7 kcal/kg FFM) [10].

A study by Lieberman et al. [2] also confirmed the effect of energy availability on menstrual disorders. A decrease of 1 unit in energy availability was associated with a 9% increase in the likelihood of menstrual disorders. Even a 10-unit reduction in energy availability, from 38 kcal/kg FFM/day to 28 kcal/kg FFM/day, which could be caused, for example, by an intervention aimed at weight reduction, was sufficient to significantly reduce the frequency of LH secretion and increase the likelihood of a luteal phase defect [14].

Table 1 summarizes the key results from the studies discussed above to better illustrate these findings.

These findings highlight the importance of adequate energy availability for menstrual cycle regulation and reproductive health in women. Energy availability strongly correlates with the frequency of LH pulses, their amplitude, and the incidence of menstrual disorders [1]. A study by Koltun et al. confirmed this correlation by finding that a decrease in energy availability by 1 unit (kcal/kgFFM/day) reduced LH pulse frequency by 0.017 pulses/hour. It was also shown that for every 0.1 unit decrease in LH pulse frequency, the chances of developing a luteal phase defect were 22 times higher than with an optimal ovulatory cycle. The frequency of LH pulses in cycles with a luteal phase defect averaged 49% of the frequency in cycles with no menstrual disorders [14]. A study by Lieberman et al. [2] also confirmed the effect of energy availability on menstrual disorders. A decrease of 1 unit in energy availability was associated with a 9% increase in the likelihood of menstrual disorders. Even a 10-unit reduction in energy availability, from 38 kcal/kg FFM/day to 28 kcal/kg FFM/day, which could be caused, for example, by an intervention aimed at weight reduction, was sufficient to significantly reduce the frequency of LH secretion and increase the likelihood of a luteal phase defect [14]. A study by Williams et al. [16] focused on assessing the impact of different levels of energy deficit resulting from combining a low-calorie diet with intense exercise on menstrual function. This study included untrained, regularly menstruating women aged 18 to 30 years with normal BMIs and average body fat levels [15].

Study participants were randomly assigned to one of four groups: an exercise control group and three exercise energy deficit (ED) groups of varying degrees: mild (ED1: −8 ± 2%), moderate (ED2: −22 ± 3%), and severe (ED3: −42 ± 3%). Thirty-four people completed the study. Weight loss was −3.8 ± 0.2 kg in the mild deficit group, −2.8 ± 0.6 kg in the moderate deficit group, −2.6 ± 1.1 kg in the severe deficit group, and was minimal in the control group at −0.9 ± 0.7 kg. Most weight loss was due to fat reduction, and no significant changes in lean body mass were observed. T3 (triiodothyronine) and IGF-1 (insulin-like growth factor) concentrations decreased significantly in all exercise groups compared to baseline values but not in the control group [15].

The incidence of menstrual disorders increased proportionally to the duration of the intervention and proportionally to the size of the deficit, with the lowest percentage of patients in the control group (13%) and the highest percentage of patients in the severe deficit group (88%). These results indicate the negative impact of an energy deficit caused by a low-calorie diet and intense exercise on menstrual function and reproductive health in women [15].

According to the results of the study [15], an energy deficit of −22 to −42% compared to baseline energy supply, i.e., −470 to −810 kcal/day, is sufficient to induce clinical and subclinical menstrual disorders. Subclinical menstrual disorders are subtle ovulatory disturbances, such as anovulatory cycles or short luteal phases, occurring despite regular menstrual cycles and adequate estrogen levels, resulting in lower progesterone levels and potentially impacting fertility and overall health [17]. If these disorders occur over a long period, they can lead to the disappearance of menstruation. In the study, there were no significant changes in the length of the menstrual cycle or the follicular phase of the participants, but there was a shortening of the luteal phase. This means that there were no visible symptoms of menstrual disruption. Therefore, women exercisers may not be aware of such disorders or their potential impact on fertility and bone health, which can be detected by monitoring urinary metabolites of menstrual cycle hormones or through blood tests [15]. In addition, the study found that the mean percentage energy deficit was a strong predictor of the incidence of menstrual disorders, while weight loss, changes in body fat, or changes in RMR (Resting Metabolic Rate) were not significantly associated with these disorders [15].

Another study, which included 25 women participating in elite endurance events, found that women with menstrual disorders had 19% lower fat mass compared to women with a regular menstrual cycle, despite no differences in the amount of training performed. There were also no significant differences in energy expenditure between the groups, but women with menstrual disorders had a lower ratio of actual RMR to predicted RMR. The study showed that women with menstrual disorders stayed in a catabolic state (i.e., an energy deficit) for a more extended period during the day, with an energy balance below 0 and −300 kcal, compared to women with a regular menstrual cycle. Regression analysis showed that the more hours spent in an energy deficit of less than 0 kcal and less than −300 kcal, the lower the ratio of actual RMR to predicted RMR, lower estrogen levels, and higher cortisol levels [18].

Additionally, it was noted that participants with menstrual disorders consumed more meals and snacks per day compared to regular menstruating women. However, there was no correlation between meal frequency and kilocalorie provision, but time spent in an energy deficit was positively correlated with meal frequency. An interesting observation was that despite similar energy availability and energy balance throughout the day in both groups, athletes with menstrual dysfunction spent more time in a glucose-deficient state, negatively affecting the hypothalamic–pituitary–gonadal axis function [17].

The study also found that time spent in energy deficit during the day was associated with higher cortisol levels and lower levels of estrogen and thyroid hormone (T3), which may suggest the presence of a state of biological stress. LH pulses, T3 levels, and cortisol levels depend on glucose availability in the brain. Prolonged periods of energy deficit during the day may adversely affect hormonal changes [13].

To date, it has not been possible to determine conclusively whether exercise alone can affect hormone regulation, as most studies mainly focus on generating an energy deficit. However, it should be emphasized that physical training can also be a stressor for the body [19].

This connection between exercise and hormonal suppression was further exemplified in the study by Williams et al. [15]. This study observed a significant decrease in critical hormones such as estrone-1-glucuronide (E1G) and pregnanediol 3-glucuronide (PDG), which are indicators of estradiol and progesterone levels, and luteinizing hormone (LH). Interestingly, these hormonal changes were observed across all groups regardless of differences in energy availability (EA), suggesting that physical training alone might have a suppressive effect on sex hormone concentrations independent of energy availability [2,15]. However, it should be noted that further studies confirming this observation are lacking.

Finally, the scientific literature generally accepts that a crucial factor leading to menstrual disorders in women is low energy availability, i.e., an insufficient supply of adequate energy to the body [20]. These findings highlight the importance of proper energy availability for menstrual cycle regulation and reproductive health in women.

### 3.2. Dietary Intervention

Optimal energy availability is crucial for proper reproductive system functioning, yet the precise caloric intake required to restore menstrual function remains unclear. Studies by De Souza et al. [21], Łagowska et al. [22], and others have examined the impact of increased caloric intake on menstrual recovery in women with secondary amenorrhea and oligomenorrhea, focusing on athletes and ballet dancers. The study by De Souza et al. study [20] involved 33 women aged 18–35 with secondary amenorrhea or oligomenorrhea, a BMI of 16–25 kg/m^2^, and who were exercising more than two hours per week. Over 12 months, 17 participants gradually increased their caloric intake by 20–40%, while 16 maintained their usual intake. The intervention group saw an average increase in caloric intake of 330 ± 65 kcal/day compared to a change of −66 ± 68 kcal/day in the control group. The intervention group experienced a weight gain of 2.6 ± 0.4 kg after 12 months, while the control group’s average change was 0.7 ± 0.4 kg. Additionally, fat mass and body fat percentage increased by 2.0 ± 0.3 kg and 2.7 ± 0.4%, respectively. Notably, T3 concentration in the intervention group increased by 9 ± 4 ng/dL. These changes improved menstrual regularity, demonstrating that a slight caloric surplus of approximately 300–350 kcal/day was sufficient for restoring menstrual cycles.

The Łagowska et al. [21] study focused on 52 well-trained female athletes and ballet dancers with menstrual disorders, who all trained more than four times per week. Over nine months, participants increased their caloric intake by 20–30%. Initially, both groups showed lower-than-normal serum leptin concentrations. After nine months, energy availability exceeded 30 kcal/kg FFM/day. In the group of ballet dancers, body weight increased by approximately 1.3 kg, while athletes did not show significant changes in weight, BMI, or body composition. However, LH concentrations and the LH to FSH ratio improved. At the study’s start, five dancers and five athletes were diagnosed with functional hypothalamic amenorrhea (FHA). By the end, three dancers and seven athletes had regained their menstrual cycles. These hormonal improvements suggest that increased caloric intake is critical in recovering menstrual function.

Additional research by De Souza et al. [23] also assessed the impact of increased energy intake on bone health and menstrual function in exercising women with menstrual disorders in a randomized controlled trial. Regardless of group assignment, all participants received daily calcium and vitamin D3 supplements to ensure a daily intake of 1200 mg of calcium and 400 IU of vitamin D3. The study used DEXA (bone densitometry test) scans to assess body composition, including fat mass [kg], lean mass [kg], and bone mineral density (BMD) [g/cm^2^]. These scans were performed at baseline and at months 6 and 12 of the study. BMD assessment included the whole body, the lumbar spine (L1-L4), and the femur (femoral neck and total hip).

The study showed that both groups experienced a reduction in femoral neck BMD at months 6 and 12 and a decrease in total hip BMD at month 6. For example, the Oligo/Amen+Cal group (women with oligomenorrhea or amenorrhea who received additional kilocalories as part of the study’s intervention) saw a decrease in femoral neck BMD of approximately 0.87% from baseline to month 12. In comparison, the Oligo/Amen Control group (women with oligomenorrhea or amenorrhea who did not receive the additional calorie intervention and continued their regular diet and routine) experienced a decrease of about 1.28% in the same period. The total hip BMD also declined slightly in both groups. This suggests that despite the increased energy intake, both groups still experienced adverse effects on bone health. It was concluded from the study that despite the rise in dietary kilocalories, and thus dietary energy availability, as well as increases in body weight, body fat, and menstrual recovery, bone mineral density did not improve significantly in either group. This outcome may be due to the study’s relatively short duration (12 months) and the possibility that the increase in dietary kilocalories (approximately 352 kcal/day) was insufficient to induce positive changes in BMD in response to improved energy or reproductive status. A more extended follow-up period and potentially higher energy intake might be necessary to observe significant improvements in bone health. A more extended follow-up period and potentially higher energy intake might be required to observe significant improvements in bone health.

Mallinson et al. [24] described cases of two exercising women with FHA of different durations (short-term and long-term) and compared their recovery as a result of a 12-month nutritional intervention. The 19-year-old participant with long-term FHA required a significant weight gain (4.2 kg) to achieve regular menstrual cycles, while the 24-year-old with short-term FHA regained ovulatory cycles with a smaller weight gain (2.8 kg). Both participants showed improvements in leptin and T3 concentrations, reinforcing the importance of these hormones in menstrual recovery. However, persistent menstrual irregularities and the need for individualized approaches were noted.

A study by Cominato et al. [25] involving adolescents with eating disorders showed significant improvements in nutritional status and hormone levels over 20 weeks. The recovery of menstrual function was linked to increases in BMI, LH, IGF-1, and estradiol. Other hormones measured in the study were FSH, prolactin, GH, TSH, and thyroid hormones, but they did not show significant changes between the beginning of the research and the end of the intervention. Notably, IGF-1 levels were particularly substantial in distinguishing those who regained menstruation from those who did not, suggesting it as a potential marker for recovery. For women who regained their menstrual cycle, the mean IGF-1 concentration at the end of the study was 454.0 ± 7.9 ng/mL, and among women who did not regain their menstruation, 367.4 ± 133 ng/mL [24].

Evidence suggests that the loss of menstrual function is not solely linked to body mass index (BMI) and can occur in women with average or higher BMIs. However, a study conducted by Daempfle et al. [26] focused on the relationship between BMI and the recovery of regular menstruation by achieving an appropriate body weight. This study included 152 girls aged 11–18 with diagnosed eating disorders and who were underweight. Among these participants, 47% spontaneously regained menstrual function during the observation period, while the rest did not.

The study found a strong correlation between the percentage of expected body weight achieved (%EBW, where expected body weight (EBW) was adjusted for age, and %EBW was calculated as observed BMI/EBW × 100) and the resumption of menstruation within 12 months of observation. A reduction in BMI by about 1 kg/m^2^ doubled the risk of amenorrhea. The average %EBW among patients who spontaneously regained menstruation within 12 months was 91% EBW, while participants who did not regain menstrual function had an average body weight of approximately 86% EBW. The study also discovered that patients with higher pre-morbid BMI or %EBW (higher BMI at the onset of menstruation loss) were less likely to recover regular menstrual cycles during the observation period. This suggests a significant difference between expected body weight and the weight at which menstruation ceased. This study confirms that BMI is not a reliable predictor of the return of menstrual function [25]. The findings from these studies are summarized in Table 2.

These studies collectively indicate that even modest increases in caloric intake can significantly impact menstrual recovery in exercising women. Increased energy availability led to improved metabolic status, weight gain, and hormonal changes necessary for menstrual function restoration. Specifically, the De Souza et al. [20] study highlighted a positive correlation between fat mass gain and menstrual recovery, with increased T3 concentration and improved metabolism observed in the intervention group.

The Łagowska et al. [21] study found that menstrual recovery correlated with increased body fat and leptin concentrations. Significant hormonal improvements, particularly in LH concentration, were notable, especially among those who regained menstrual cycles. This suggests that leptin and T3 could be potential biomarkers for monitoring menstrual recovery.

In conclusion, increased caloric intake can effectively restore menstrual function in exercising women with menstrual disorders. These findings underscore the importance of energy availability in reproductive health and suggest that non-pharmacological interventions, such as dietary modifications, are beneficial. Future research should focus on the long-term effects of increased caloric intake on bone health and metabolic markers.

### 3.3. The Role of Micronutrients in the Management of FHA

While the primary focus of FHA treatment is increasing caloric intake and macronutrient balance, addressing micronutrient deficiencies is also critical. Proper vitamin D3 and calcium ion supply is crucial for bone metabolism and fertility. While decreased 25-(OH)D levels have been associated with an increased risk of menstrual irregularities, such as long follicular and short luteal phases, this relationship may be influenced by other factors, such as obesity, which is independently associated with both low vitamin D levels and conditions like PCOS. However, vitamin D supplementation might be beneficial in restoring menstrual function in women with functional hypothalamic amenorrhea (FHA) [27]. The study by Singh et al. [28] provides valuable insights into the potential role of vitamin D in influencing menstrual cycle regularity and, by extension, functional hypothalamic amenorrhea (FHA). Lower vitamin D levels may result in decreased estrogen production due to impaired follicular development. This hypoestrogenic state is a hallmark of FHA and leads to various clinical manifestations, including amenorrhea, low bone mineral density, and reproductive issues [26,27,29]. Despite the limited evidence from well-powered randomized controlled trials directly supporting vitamin D supplementation as a treatment for FHA, understanding the role of vitamin D in the body and its influence on factors contributing to FHA suggests that supplementation may be beneficial. Furthermore, chronic stress can lead to magnesium depletion, which may exacerbate the stress response. In FHA, stress is a known factor that disrupts the normal secretion of gonadotropin-releasing hormone (GnRH), leading to amenorrhea. Magnesium deficiency, which is prevalent under stress conditions and poor nutritional intake, could contribute to FHA. Ensuring adequate magnesium intake or supplementation may help alleviate some of the stress-related hormonal imbalances seen in FHA [30,31,32]. Although there is a lack of strong evidence specifically confirming the effectiveness of magnesium supplementation in FHA, the known benefits of magnesium in reducing stress and supporting hormonal balance indicate that it may be a useful component of treatment. Despite not experiencing regular menstrual blood loss, women with functional hypothalamic amenorrhea (FHA) often suffer from iron deficiency. This deficiency is attributed mainly to insufficient dietary intake and decreased absorption of iron due to high consumption of fiber and phytic acid [17,33,34].

The study by Tian and Diaz [35] highlights the crucial role of zinc in maintaining meiotic arrest and oocyte development, finding that zinc deficiency leads to premature oocyte maturation and impaired ovulation. This is particularly relevant to Functional Hypothalamic Amenorrhea (FHA), a condition where stress-induced hormonal imbalances disrupt the hypothalamic–pituitary–ovarian axis. Zinc deficiency may worsen these imbalances, further contributing to reproductive dysfunction. Including zinc supplementation in FHA management could improve ovarian function and restore menstrual cycles. Additionally, zinc’s role in reducing cortisol levels, inflammation, and oxidative stress underscores its potential therapeutic value [36]. However, while direct evidence supporting zinc supplementation in FHA is still emerging, based on its known physiological effects and findings from related studies [35,36], incorporating zinc supplementation could be considered a supportive measure in managing FHA.

### 3.4. Psychological Interventions in FHA

Additionally, psychological treatment is a crucial aspect of managing FHA. Cognitive–behavioral therapy (CBT) has been shown to benefit women with FHA, helping to alleviate resistance to dietary and lifestyle changes. For women with FHA, increasing body weight and caloric intake can be particularly challenging due to a variety of physiological and psychological factors. Just as patients with obesity find it difficult to eat less due to genetic and hormonal appetite regulation, women with FHA may struggle against appetite pathways. Studies have shown that modifying dietary intake requires tailored strategies and psychological support [36,37]. A study by Berga et al. [37] examined whether cognitive–behavioral therapy (CBT) could restore ovarian function in women with functional hypothalamic amenorrhea (FHA). This randomized, controlled trial involved sixteen women divided into two groups: one receiving CBT and the other under observation for 20 weeks. Results showed that 87.5% of women in the CBT group resumed ovarian activity compared to 25% in the observation group, indicating that CBT is an effective non-pharmacological treatment for FHA by addressing stress and maladaptive attitudes [37]. Such therapeutic interventions address underlying issues like eating disorders and perfectionism, which are often associated with FHA [37,38].

It is also recommended that subtle modifications be made to training volume and intensity, ensuring adequate recovery rather than drastic changes. Alternative forms of exercise, such as yoga, walking, or light cycling, can reduce stress without significantly compromising the patient’s psychological well-being [39,40,41]. Studies have shown that in response to a stress challenge, there is a substantially more significant increase in cortisol and a decrease in blood glucose in women with FHA compared to healthy women. High-intensity exercise, such as sprinting or weightlifting, can lead to greater cortisol output than low-intensity exercise, such as walking or yoga [40,41]. Cortisol can inhibit the production of GnRH by the hypothalamus and, thus, the release of LH and FSH from the pituitary gland, which can disrupt ovulation and the menstrual cycle. Studies in monkeys have shown that a single stressor rarely causes menstrual disorders but rather results from the coexistence of different stressors [42,43].

The primary treatment for athletes is to increase the caloric content of their diets rather than modify their training, as they cannot afford to do so. However, recreational athletes who want to restore menstrual function as soon as possible should increase their kilocalorie supply while reducing exercise intensity. It is essential to remember that a complete ban on physical activity can be stressful, especially when accompanied by eating disorders and perfectionism. Therefore, working with a psychotherapist to help resistance to change is necessary. Subtle modifications to training volume, reduced exercise intensity, and ensuring adequate recovery are recommended rather than making drastic changes. Alternative forms of training, such as yoga, walking, or light cycling, can also be considered [18,38,39,40,41,42].

### 3.5. Other Nutritional and Lifestyle Interventions

In treating FHA, a holistic approach and fluid and comprehensive changes are essential to prevent the body from prolonging its pathological state [5]. As FHA treatment aims to restore energy balance and provide the body with sufficient kilocalories to support physiological functions, patients’ nutritional status and eating habits must be assessed [4,6,7,44].

Given patient variability, continuous monitoring of dietary caloric intake is necessary to restore menstrual function. A critical factor in this process is energy availability (EA), with evidence showing that the risk of menstrual disorders increases as EA decreases. [14]. Although EA calculations can help predict menstrual disorders, they must be adjusted based on dietary history, attitudes toward food, and weight change history. Repeated EA measurements are recommended to observe trends and establish appropriate target values for each individual [45,46].

Specific ranges of EA have been accepted in the literature [1,47,48,49]. Low EA is <30 kcal/kg FFM/day, equivalent to basal metabolism, below which many physiological disorders can occur. A range of 30–45 kcal/kg FFM/day is considered reduced energy availability and is only recommended for short periods, e.g., during fat reduction. A standard EA value of≥ 45 kcal/kg FFM/day provides energy balance. Therefore, an initial increase in energy availability to 45 kcal/kg FFM is usually recommended in female athletes. However, the energy consumed for physical activity should also be considered [50,51].

Although the number of kilocalories required to restore menstrual function remains uncertain, studies have shown that gradually increasing dietary caloric content in women with FHA increases the likelihood of restoring the menstrual cycle or increasing LH and FSH levels, which is positively correlated with increased energy availability [20,21,22].

Nutritional interventions should also consider the timing of energy intake. Energy redistribution over 24 h can help to avoid latent deficits, as prolonged intervals between meals lead to significant glucose drops. Glucose availability in the brain is crucial for the regulation of luteinizing hormone (LH) pulses and the concentration of T3 and cortisol [17].

A study by Melin et al. utilized the Low Energy Availability in Females Questionnaire (LEAF-Q) to evaluate dietary patterns among 84 female athletes aged 18–39 years, who all trained at least five times per week. This questionnaire was specifically designed to identify those at risk for the Female Athlete Triad (Triad). The findings revealed that athletes with functional hypothalamic amenorrhea (FHA) consumed significantly lower amounts of dietary fats and carbohydrates compared to their counterparts without FHA. Conversely, these athletes exhibited a higher intake of dietary protein and fiber [52]. An adequate supply of carbohydrates in the diet is essential, as they are the body’s primary and fastest energy source, and a low supply of carbohydrates is correlated with glycogen depletion [53,54]. An adequate fat supply is also essential, mainly because of the sources of unsaturated fatty acids and their beneficial effects on inflammation, the nervous system, and fertility [55]. A consequence of dietary fat deficiency is also the risk of deficiency of fat-soluble vitamins (A, D, E, K). An inadequate amount of dietary fat interferes with the central nervous system, mainly due to the regulation of neurotransmission by fatty acids and their metabolites, thereby affecting emotional behavior [17,56]. Moreover, women with Functional Hypothalamic Amenorrhea (FHA) who are also engaged in sports typically have high caloric needs, which can be challenging to meet. In such cases, while prioritizing a diet based on whole foods is essential, incorporating processed foods can facilitate meeting their caloric requirements. Examples of beneficial processed foods include protein bars, fortified cereals, and energy-dense snacks like nut butter or dried fruits. Ensuring an adequate caloric intake is paramount, and these processed options can help bridge the gap to support overall health and recovery [53,57].

It is recommended that the mainstay of intervention in FHA is non-pharmacological treatment. However, in cases where non-pharmacological treatments for FHA are insufficient and bone fractures occur, hormone replacement therapy (HRT) is preferred over contraceptives for bone protection. HRT, as recommended by the Endocrine Society, is favored due to its beneficial effects on insulin-like growth factor 1 (IGF-1) and its role in bone metabolism [49].

## 4. Discussion

The findings of this review highlight the significant role of energy availability and its intricate relationship with reproductive health, particularly in the context of Functional Hypothalamic Amenorrhea (FHA). FHA, characterized by the absence of menstruation, is a condition closely associated with energy deficiency, excessive physical activity, and psychological stress. This review underscores that the primary factor in the development and persistence of FHA is low energy availability, which directly impacts the pulse frequency of gonadotropin-releasing hormone (GnRH) and subsequently luteinizing hormone (LH) and follicle-stimulating hormone (FSH), leading to disrupted ovarian function [2,3,4].

The data suggest that restoring energy balance through increased caloric intake and moderate physical activity is essential for the recovery of menstrual function in women with FHA [21,33]. The studies reviewed show that an energy availability threshold of 30 kcal/kg FFM/day is often cited as critical. Yet, variations exist in individual responses, indicating that some women may require higher energy availability to restore regular menstrual cycles. This highlights the necessity of personalized treatment approaches in managing FHA, as the optimal energy requirements may vary based on individual differences in metabolism, body composition, and exercise habits [10,11,12,13,14,15].

Furthermore, the importance of body composition, particularly body fat percentage, is evident. The review indicates that an increase in body fat percentage above 22% may be necessary for resuming menstruation, reinforcing the idea that body composition, rather than just body weight, is crucial in evaluating and treating FHA. This aligns with findings that even small increases in body fat can significantly improve the likelihood of menstrual recovery [24].

Micronutrient intake also plays a critical role in managing FHA. Adequate levels of vitamin D, calcium, magnesium, and zinc are essential for bone health and supporting hormonal balance and stress management. The review points out that deficiencies in these micronutrients can exacerbate the hormonal imbalances associated with FHA, suggesting that nutritional interventions should include caloric adjustments and a focus on micronutrient sufficiency [30,31,32].

The review also addresses the psychological dimensions of FHA, advocating for the integration of cognitive–behavioral therapy (CBT) as part of the treatment plan. This approach is particularly relevant for individuals whose FHA is compounded by psychological stress, disordered eating, or perfectionism. CBT can help patients develop healthier attitudes towards food, exercise, and body image, which are critical for sustained recovery [35].

The review suggests that modifying physical activity is crucial in addition to dietary and psychological interventions. However, it cautions against the complete cessation of exercise, especially for athletes, as this could lead to additional stress. Instead, reducing exercise intensity and volume while ensuring adequate caloric intake might be more effective in restoring menstrual function without causing additional psychological stress [33,35,38,39,40].

Despite the insights provided, the review acknowledges the limitations of current research, the variability in response to treatment, and the need for more long-term studies to understand the full impact of dietary and lifestyle interventions on FHA recovery. Future research should focus on refining energy availability guidelines and exploring the interactions between diet, exercise, and psychological factors in different populations of women affected by FHA.

## 5. Conclusions

In conclusion, this review highlights the complex interplay between energy availability, body composition, micronutrient intake, and psychological factors in the management of FHA. A multidisciplinary approach, including personalized dietary adjustments, psychological support, and careful physical activity management, is essential for the effective treatment of this condition. Such an approach addresses the immediate symptoms of FHA and promotes long-term reproductive and overall health in affected women.

## Figures and Tables

**Table 1:** Summary of Studies on the Impact of Energy Availability on the Menstrual Cycle.

Study	Energy Availability (EA) Levels	Findings on LH Pulses	Findings on Menstrual Disorders	Notes
Loucks et al. [1]	30 kcal/kg FFM/day 20 kcal/kg FFM/day 10 kcal/kg FFM/day	−20 kcal/kg FFM/day, 16% decrease in LH pulse frequency, a 21% increase in amplitude, −10 kcal/kg FFM/day, 39% decrease in LH pulse frequency, 109% increase in amplitude	EA below 30 kcal/kg FFM/day is linked to a higher likelihood of menstrual disorders, such as oligo/amenorrhea	Establishes a threshold for EA below which LH pulsatility and menstrual function are impaired
Koltun et al. [14]	No specific threshold identified. Reduction from 38 to 28 kcal/kg FFM/day	Decrease in LH pulse frequency by 0.017 pulses/hour for each unit decrease in EA. Lower EA also significantly reduces LH secretion frequency	Increased risk of luteal phase defects with lower EA. Significant EA reductions heighten the likelihood of menstrual disorders	No clear threshold for EA, but findings suggest more severe impacts with greater EA reduction
Liberman et al. [2]	EA < 30 kcal/kg FFM/day	LH pulse frequency decreases and amplitude increases with reduced EA	Menstrual disorders (luteal phase defects, anovulation, oligomenorrhea) become more likely as EA decreases but can occur even above 30 kcal/kg FFM/day	Highlights that menstrual disorders can occur even above 30 kcal/kg FFM/day, challenging the strict threshold concept
Reed et al. [10]	FHA group: 30.9 ± 2.4 kcal/kg FFM/day vs. 36.9 ± 1.7 kcal/kg FFM/day in control	No specific findings on LH pulses were provided	Women with functional hypothalamic amenorrhea (FHA)had significantly lower EA compared to regularly menstruating women	EA of 30 kcal/kg FFM/day does not clearly differentiate between regular menstruation and menstrual disorders

Abbreviations: EA—Energy Availability, FFM—Fat-Free Mass, LH—Luteinizing Hormone, FHA—Functional Hypothalamic Amenorrhea.

**Table 2:** Summary of Studies on the Impact of Increased Caloric Intake on Menstrual Recovery.

Study	Population	Intervention	Results	Conclusion
De Souza et al. [20]	Thirty-three women (age 18–35) with secondary amenorrhea or oligomenorrhea, BMI 16–25 kg/m^2^, exercising >2 h/week	Increased caloric intake by 330 ± 65 kcal/day (20–40%) over 12 months	Weight gain: 2.6 ± 0.4 kg, Fat mass gain: 2.0 ± 0.3 kg, Increase in T3 concentration by 9 ± 4 ng/dL	A modest caloric surplus (~300–350 kcal/day) is sufficient for restoring menstrual cycles. Improved energy balance leads to menstrual recovery
Łagowska et al. [21]	Fifty-two athletes and ballet dancers with menstrual disorders, training >4 times/week	Increased caloric intake by 20–30%, energy availability increased by >30 kcal/kg FFM/day over 9 months	Weight gain: 1.3 kg (ballet dancers), no significant weight changes (athletes), Increased LH and LH/FSH ratio, Menstrual recovery in 3 dancers and 7 athletes	Increased caloric intake is critical for hormonal improvement and menstrual recovery. Menstrual function can be restored when body fat mass reaches 22%
Mallinson et al. [23]	Two women with FHA of different durations	A 12-month nutritional intervention with individualized caloric increases	Weight gain: 4.3 kg (long-term FHA) and 2.8 kg (short-term FHA), Improvements in leptin and T3 concentrations	Weight gain and improved hormone levels are crucial for menstrual recovery, with individual variations of response
Cominato et al. [24]	Adolescents with eating disorders	A 20-week nutritional intervention	Recovery of menstrual function linked to increases in BMI, LH, IGF-1, and estradiol	IGF-1 may serve as a potential marker for menstrual recovery. Nutritional rehabilitation is a key to restoring menstrual function
Deampfle et al. [25]	One hundred and fifty-two girls (age 11–18) with eating disorders and underweight	Observational study followed participants over 12 months	Forty-seven percent regained menstrual function, Strong correlation between %EBW and resumption of menstruation	Achieving expected body weight is strongly associated with menstrual recovery. BMI is not a reliable predictor of menstrual function

Abbreviations: BMI—Body Mass Index, T3—Triiodothyronine, LH—Luteinizing Hormone, FSH—Follicle-Stimulating Hormone, FFM—Fat-Free Mass, FHA—Functional Hypothalamic Amenorrhea, IGF-1—Insulin-Like Growth Factor 1, %EBW—Percentage of Expected Body Weight.

## Data Availability

Data are contained within the article.
